# The Transition to Paschen’s Law for Microscale Gas Breakdown at Subatmospheric Pressure

**DOI:** 10.1038/s41598-019-42111-2

**Published:** 2019-04-05

**Authors:** Amanda M. Loveless, Guodong Meng, Qi Ying, Feihong Wu, Kejing Wang, Yonghong Cheng, Allen L. Garner

**Affiliations:** 1 School of Nuclear Engineering, Purdue University, West Lafayette, Indiana, 47907 USA; 2 State Key Laboratory of Electrical Insulation and Power Equipment, Xi’an Jiaotong University, Xi’an, 710049 China; 3 School of Electrical and Computer Engineering, Purdue University, West Lafayette, Indiana, 47907 USA

## Abstract

The decrease in electronic device size necessitates greater understanding of gas breakdown and electron emission at microscale to optimize performance. While traditional breakdown theory using Paschen’s law (PL), driven by Townsend avalanche, fails for gap distance *d* $\lesssim$ 15 *μm*, recent studies have derived analytic equations for breakdown voltage when field emission and Townsend avalanche drive breakdown. This study derives a new analytic equation that predicts breakdown voltage *V*_*B*_ within 4% of the exact numerical results of a previously derived theory and new experimental results at subatmospheric pressure for gap distances from 1–25 μm. At atmospheric pressure, *V*_*B*_ transitions to PL near the product of pressure and gap distance, *pd*, corresponding to the Paschen minimum; at lower pressures, the transition to PL occurs to the left of the minimum. We further show that the work function plays a major role in determining which side of the Paschen minimum *V*_*B*_ transitions to PL as pressure approaches atmospheric pressure while field enhancement and the secondary emission coefficient play smaller roles. These results indicate that appropriate combinations of these parameters cause *V*_*B*_ to transition to PL to the left of the Paschen minimum, which would yield an extended plateau similar to some microscale gas breakdown experimental observations.

## Introduction

Gas breakdown in the presence of electric fields is desirable for generating microplasmas for combustion^1^, electric propulsion^2^, or medical and environmental applications^3–7^ and deleterious in accelerators^8^, fusion devices^9^, micro and nanoelectronics^10,11^, and pulsed power biological experiments^12^. All these scenarios require accurately predicting gas breakdown at microscale gaps or smaller; however, the standard theory for predicting gas breakdown voltage *V*_*B*_ given by Paschen’s law (PL)^13^ fails because field emission (FE), rather than Townsend avalanche (TA), drives breakdown at these scales^14,15^. Given by ${V}_{B}={B}_{p}pd/[\,\mathrm{ln}({A}_{p}pd)-\,\mathrm{ln}\,[\,\mathrm{ln}(1+{\gamma }_{SE}^{-1})]],$ where *p* is the pressure, *d* is the gap distance, *γ*_*SE*_ is the secondary electron emission coefficient, and *A*_*p*_ and *B*_*p*_ are gas constants, PL^13^ is characterized by a minimum *V*_*B*_ that occurs when $pd=\exp (1){A}_{p}^{-1}\,\mathrm{ln}(1+{\gamma }_{SE}^{-1});$ however, at microscale, this minimum vanishes or is replaced by an extended plateau^14,15^.

Several mathematical models and simulations^15–23^ have predicted *V*_*B*_ as a function of gap distance and/or pressure to demonstrate the transition to FE. Because many of these models must be solved numerically—and those that are analytic often do not fully incorporate all mechanisms to elucidate limiting behavior—more recent studies have applied matched asymptotic theory to analytically unify FE and TA for argon at atmospheric pressure^24^, any gas at atmospheric pressure^25^, and any gas at any pressure for FE or TA driven breakdown^26^. These models can also quantify the relative contributions of FE and TA to breakdown^25–27^, demonstrate the transitions between the mechanisms, and yield an analytic expression similar to traditional vacuum breakdown^27,28^. More recent simulations have shown that electrodes with multiple sharp protrusions yielded an effective PL that combined the individual PL for each protrusion^29^. While potentially contributing to the observed extended plateau, these simulations did not fully incorporate field emission. Furthermore, real electrodes, even when polished to control surface roughness, may not necessarily have distinct, well-defined, sharp tips.

Many (albeit not all) microscale gas breakdown experiments focus on breakdown at atmospheric pressure, which is critical for the aforementioned biomedical^6,7^, environmental^5^, and combustion applications^1^. However, gas breakdown plays a major role in limiting the power levels of high power microwave devices from 1 to 100 GHz^30–32^. Additionally, electric field distribution plays a critical role in low-pressure gas breakdown^33,34^. While most vacuum electronics studies focus on device failures due to space-charge limited emission^35–38^ and multipactor^39–41^, vacuum breakdown has also been examined^28^. The development of carbon nanotube systems for vacuum electron emission^42–44^ and the potential for nanoscale systems at higher pressures^45^ motivates additional characterization of electron emission experimentally at subatmospheric pressure. While some studies have assessed pressure on the order of a few torr^21^, a study detailing the impact of pressure on the intersection of the combined FE/TA breakdown regime with PL has not been performed. This letter measures breakdown voltage at microscale for several subatmospheric pressures and assesses this behavior using a universal, matched asymptotic solution^26^. As we shall show, this universal model gives the conditions under which FE and TA driven breakdown transition to PL to the *left* of the Paschen minimum, yielding the appearance of an extended plateau.

## Results

### Experimental description and results

A detailed description and block diagram of the experimental setup can be found in ref.^27^. Briefly, the experimental system consists of a nanosecond pulse generation unit, a synchronous and delay triggering unit, an *in-situ* optical imaging unit, and an electrical parameter measurement unit. We generated the nanosecond pulse by feeding DC voltage into a high voltage solid-state switch (BEHLKE HTS-50-08-UF), which delivered adjustable nanosecond pulses with a maximum amplitude up to 5 kV. Synchronous triggering was performed by a function generator (RIGOL DG3101A). We integrated an *in-situ* optical imaging unit with an optical microscope to achieve micron-scale spatial resolution and a high-speed gated ICCD camera to attain nanosecond-scale temporal resolution. A metallographic microscope (OLYMPUS BX51M) with a long work distance objective lens (50×) magnified the micron-scale test specimen. We used a high-speed gated ICCD camera (ANDOR iStar 334 T) to detect light emission during gas breakdown with a minimum gate width of 2 ns. A current coil (Pearson 6585) monitored pulse current, an attenuator probe (100:1) measured pulsed voltage, and a digital oscilloscope (LeCroy 104MXs-B) reported the signal. This letter focuses on breakdown measurements; further experimental assessments will be reported elsewhere.

**Figure 1:**
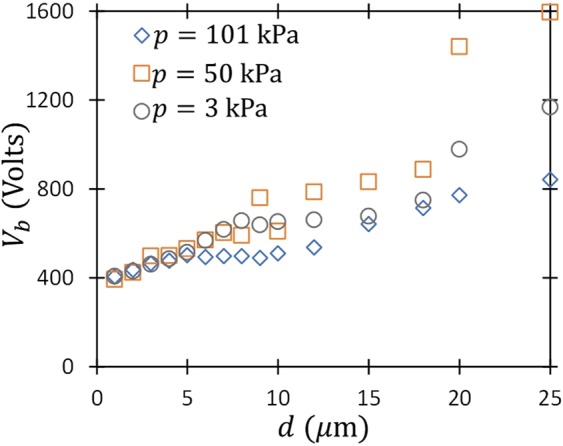
Measured breakdown voltage (*V*_*B*_) as a function of gap distance (*d*) for pressures (*p*) of 3 kPa, 50 kPa, and 101 kPa.

Figure 1 shows the experimental results for breakdown voltage in air at pressures of 3, 50, and 101 kPa for gap distances from 1–25 μm. When plotted as a function of *d, V*_*B*_ is relatively insensitive to *p* at smaller gap distances where one anticipates field emission driven breakdown. Measured *V*_*B*_ diverges with *p* for $d\gtrsim 5{\rm{\mu }}m$. While our previous theoretical studies have examined *V*_*B*_ as a function of either *p* or *d*^24–26^, the relatively large difference in *p* here suggests that collisionality, or *pd*, may elucidate the experimental behavior. Thus, we will assess *V*_*B*_(*pd*) when we apply the matched asymptotic theory to the experimental data.

### Theoretical analysis and results

We start from the general, nondimensional, universal (true for any gas) breakdown equation, given by^26^

1 $$\frac{\exp [{\bar{\varphi }}^{3/2}/(\beta \bar{E})]}{\beta {\bar{\varphi }}^{1/2}\exp ({\bar{\varphi }}^{-1/2})}\sqrt{\frac{\bar{T}\bar{E}}{\bar{p}{\bar{d}}^{2}}}\frac{\{1-{\gamma }_{SE}[\exp (\bar{\alpha }\bar{d})-1]\}}{\exp (\bar{\alpha }\bar{d})-1}=\exp (1)(1+2\bar{E}),$$

where $\bar{\varphi }=\varphi /{\varphi }_{\ast }$ is the dimensionless work function of the electrode material with ${\varphi }_{\ast }={[{(3.79\times {10}^{-4})}^{2}{B}_{FN}]}^{2}$ in eV, *β* is the field enhancement factor, $\bar{E}=E/{E}_{\ast }$ is the dimensionless breakdown electric field with ${E}_{\ast }=0.95{B}_{FN}{\varphi }_{\ast }^{3/2}$ in V/m, $\bar{p}=p/{p}_{\ast }$ is the dimensionless pressure with ${p}_{\ast }={E}_{\ast }{B}_{p}^{-1}$ in Torr, $\bar{d}=d/L$ is the dimensionless gap distance with $L={p}_{\ast }^{-1}{A}_{p}^{-1}$ in m, $\bar{T}=T/{T}_{\ast }$ is the dimensionless temperature with ${T}_{\ast }=[(\pi m{\sigma }_{CE}{B}_{p})/(8ek)]{\{{A}_{FN}/[{\varepsilon }_{0}{A}_{p}{t}^{2}(y){\varphi }_{\ast }]\}}^{2}$ in K, *γ*_*SE*_ is the secondary emission coefficient, $\bar{\alpha }=\alpha L$ is the dimensionless ionization coefficient with $\alpha ={A}_{p}p\exp (-{B}_{p}p/E)$ in m^−1^, and all terms without bars correspond to the dimensional (measured) quantities of those with bars. Additionally, *A*_*FN*_ and *B*_*FN*_ are Fowler-Nordheim constants, *A*_*p*_ and *B*_*p*_ are gas constants, *m* is the mass of the gas atom in kg, *σ*_*CE*_ isthe charge exchange cross section, *e* is the electron charge, *k* is Boltzmann’s constant, ${\varepsilon }_{0}$ is the permittivity of free space, and t^2^(*y*) ≈ 1.1^46^ is a Fowler-Nordheim correction factor used since the Schottky reduction factor, *y*, is sufficiently less than one for the data considered here. Table 1 summarizes typical values.

**Table 1:** Summary of parameters considered in this work.

Parameter	Name	Value	Unit
*V*_*B*_	Breakdown voltage	Variable	V
V_*_	Breakdown voltage scale	24.3	V
*d*	Gap distance	Variable	m
*L*	Gap distance scale	3.92 × 10^−12^	m
*E*	Breakdown electric field	Variable	V/m
E_*_	Breakdown electric field scale	6.20 × 10^12^	V/m
*p*	Pressure	Variable	kPa
p_*_	Pressure scale	1.70 × 10^8^	Torr
*T*	Temperature	300	K
T_*_	Temperature scale	7976	K
*ϕ*	Work function	4.7	eV
ϕ_*_	Work function scale	96.81	eV
*β*	Field enhancement factor	Variable	N/A
*γ*_*SE*_	Secondary emission coefficient	10^−5^	N/A

**Figure 2:**
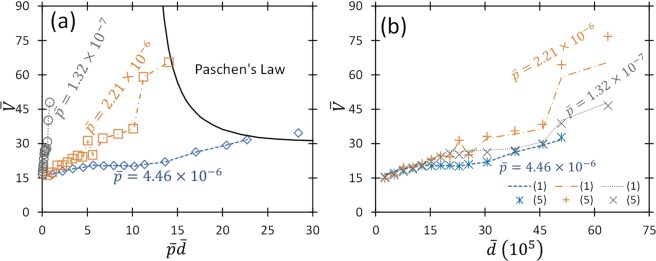
(**a**) Dimensionless breakdown voltage, $\bar{V}$, as a function of the product of dimensionless pressure and gap distance, $\bar{p}\bar{d}$, for various pressures compared to results from the universal Paschen’s law (UPL) determined from (2) with *γ*_*SE*_ = 10^−5^ using *β* from Fig. 3. The symbols represent experimental data points and the dashed lines represent the numerical solution of (1), using field enhancement factor *β* as a fitting parameter. (**b**) Dimensionless breakdown voltage, $\bar{V}$, as a function of dimensionless gap distance, $\overline{d.}\,\,$Numerical results from (1) are shown as the dashed lines and the limiting results of equation (5) are shown as symbols with *γ*_*SE*_ = 10^−5^ using *β* from Fig. 3. There is an average percent difference between equations (1) and (5) of 3.71%.

**Figure 3:**
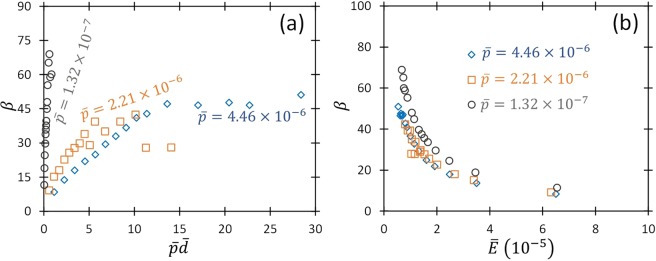
(**a**) Field enhancement factor, *β*, as a function of the product of the dimensionless pressure and gap distance, $\bar{p}\bar{d}$, obtained by fitting the experimental data from Fig. 2. (**b**) Field enhancement factor, *β*, as a function of the dimensionless electric field, $\bar{E}$.

We numerically solve Equation (1) and choose *β* to fit to the nondimensionalized experimental data from Fig. 1 as a function of $\bar{p}\bar{d}$, with *γ*_*SE*_ = 10^−5^ and $\bar{V}=\bar{E}\bar{d}$. Figure 2a shows the fitting of Equation (1) and the universal PL (UPL)^26^, given by

2 $${\bar{V}}_{B}=\frac{\bar{p}\bar{d}}{\mathrm{ln}(\bar{p}\bar{d})-\,\mathrm{ln}[\mathrm{ln}(1+{\gamma }_{SE}^{-1})]},$$

to the measured data with *β* shown in Fig. 3. We note that the experimental data for 50 kPa and 101 kPa actually intersects with the UPL, indicating the transition from the combined FE/TA regime to the traditional PL. Moreover, the 50 kPa data intersects the UPL to the left of the Paschen minimum, while the 101 kPa data intersects the UPL near the minimum, as observed in previous applications of this theory to atmospheric pressure data^26,27^. This stands to reason since previous results^47^ indicate the transition should occur around 18 μm. Since the curves in Fig. 2a are universal, they hold for any combination of parameters that yield these intersections, so the intersection with the UPL could occur on either side of the minimum at atmospheric pressure depending upon gas and electrode conditions.

We can analytically assess this intersection since $\bar{\alpha }\bar{d}\gg 1$ generally for the data considered here, allowing us to simplify $\bar{V}$ to obtain^26^

3 $$\bar{V}=\frac{\bar{d}}{{{\rm{\Lambda }}}_{2}}[-{{\rm{\Delta }}}_{2}-\sqrt{{{\rm{\Delta }}}_{2}^{2}-2{{\rm{\Lambda }}}_{2}({\bar{\varphi }}^{3/2}/\beta )}],$$

where

4 $$\begin{array}{rcl}{{\rm{\Delta }}}_{2} & = & \frac{\mathrm{ln}[\bar{T}{\bar{p}}^{-1}{\bar{d}}^{-2}]}{2}-\,\mathrm{ln}[\beta {\bar{\varphi }}^{1/2}]-{\bar{\varphi }}^{-1/2}-\frac{\mathrm{ln}[{{\rm{\Lambda }}}_{2}]}{2}\\  &  & -\,\mathrm{ln}[\exp (\frac{\bar{p}\bar{d}}{\exp (1)})-1]+\,\mathrm{ln}\{1-{\gamma }_{SE}[\exp (\frac{\bar{p}\bar{d}}{\exp (1)})]\}-\frac{3}{2},\end{array}$$

and Λ_2_ = 1 × 10^5^. Analogous to ref.^27^, we can further simplify (3) to obtain a limiting equation for $\bar{V}$, given by

5 $$\bar{V}=\frac{{\bar{\varphi }}^{3/2}}{\beta |{{\rm{\Delta }}}_{2}|}\bar{d}.$$

Figure 2b compares the limiting results from equation (5) to the numerical calculations from equation (1) using *β* from Fig. 3. The limiting results agree well with equation (1) at low $\bar{p}\bar{d}$ and deviate as $\bar{p}\bar{d}$ increases. The numerical results of equation (1) and analytic results of equation (3) have an average percent difference of 3.97% while the results of equation (1) and the limiting results of equation (5) differ by an average of 3.71%. Thus, we use equation (5) in Fig. 2b and the remainder of the analytic assessment without sacrificing accuracy. Also important concerning global universality, Fig. 2 emphasizes that the breakdown voltage scales differently in the different regimes. Upon satisfying the PL condition (transitioned from the FE/TA combined regime to the conventional PL regime), the behavior the breakdown voltage scales with *pd* and one recovers the UPL. At smaller gaps, Fig. 2a shows that breakdown voltage scales with $\bar{d}$. Thus, while breakdown exhibits universal behavior, the scaling of  this universal behavior varies depending upon the dominant breakdown mechanism.

Figure 3 shows *β* for fitting equation (1) to the data in Fig. 2 as functions of the product of dimensionless pressure and gap distance, $\bar{p}\bar{d}$, and the dimensionless electric field, $\bar{E}$. For 50 kPa and 101 kPa, *β* increases linearly with increasing $\bar{p}\bar{d},$ as observed previously in the FE dominant regime at atmospheric pressure^26,27^. Eventually, *β* approaches a constant, which corresponds to the transition from the FE to TA regimes, as also observed previously^26,27^. Interestingly, this occurs at a lower $\bar{p}\bar{d}$ for 50 kPa. Previous results indicate that this transition is not solely driven by $\bar{p}\bar{d}$, but by $\bar{p}$ and $\bar{d}$ independently, which is supported by this work. For 3 kPa, *β* also increases linearly at low $\bar{p}\bar{d},$ but much more rapidly. While the current experiments cannot achieve sufficient voltage to measure *V*_*B*_ at larger *d* for 3 kPa, the theory suggests that the intersection with the UPL will occur at a much higher *β* than either of the other pressures studied. Figure 3b indicates that *β* is a function of $\bar{E}$, which is also supported by previous work^48,49^. Interestingly, *β* at the two highest pressures studied here is identical when plotted as a function of $\bar{E},$ suggesting potential universality in this regime. Future work at lower pressures and larger gap distances can better characterize these transitions and further characterize the dependence of *β* on $\bar{E}$.

**Figure 4:**
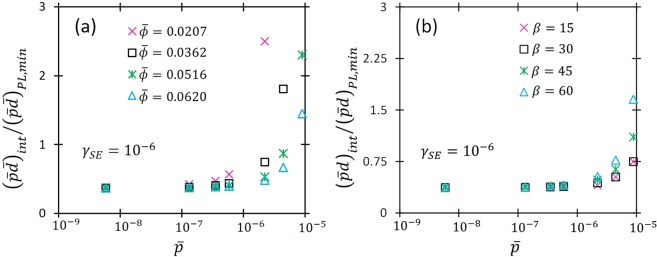
The ratio of the product of dimensionless pressure and gap distance, $\bar{p}\bar{d}$, causing the transition to Paschen’s law, ${(\bar{p}\bar{d})}_{int}$, to $\bar{p}\bar{d}$ corresponding to the Paschen minimum, ${(\bar{p}\bar{d})}_{PL,min}$, as a function of $\bar{p}$ for various values of (**a**) $\bar{\varphi }$ with *β* = 60 and *γ*_*SE*_ = 10^−6^, and (**b**) *β* with *γ*_*SE*_ = 10^−5^ and $\bar{\varphi }=0.0465$.

Finally, we consider the impact of *γ*_*SE*_, *β*, and $\bar{\varphi }$, on the transition from the FE/TA regime to the UPL. Understanding how these parameters affect breakdown is vital for developing a predictive model, since *γ*_*SE*_ and *β* are difficult to determine *a priori* and the asymptotic prediction of *V*_*B*_ is very sensitive to variations in *β* and $\bar{\varphi }$ in the FE/TA regime^50^. Thus, elucidating the influence of these terms on *V*_*B*_ will clarify the transition to UPL under different *p* and *d*. The transition from the FE/TA regime to the UPL occurs when the limiting expression of equation (5) matches the UPL from equation (2), so we numerically solve

6 $$\frac{\bar{p}\beta |{{\rm{\Delta }}}_{2}|}{{\bar{\varphi }}^{3/2}\{\mathrm{ln}(\bar{p}\bar{d})-\,\mathrm{ln}[\mathrm{ln}(1+{\gamma }_{SE}^{-1})]\}}=1$$

for $\bar{d}\,\,$with a given *γ*_*SE*_, *β*, and $\bar{p}$. Figure 4 shows the ratio of $\bar{p}\bar{d}$ for the transition, ${(\bar{p}\bar{d})}_{int}$, to the value corresponding to the standard “Paschen minimum” of Equation (2) by setting $d\bar{V}/d(\bar{p}\bar{d})=0$ to give

7 $${(\bar{p}\bar{d})}_{PL,min}=\exp \{1+\,\mathrm{ln}[\mathrm{ln}(1+{\gamma }_{SE}^{-1})]\}.$$

When ${(\bar{p}\bar{d})}_{int}/{(\bar{p}\bar{d})}_{PL,min} < (\, > \,)1$, Equations (2) and (3) intersect to the left (right) of the traditional Paschen minimum. For example, at atmospheric pressure, *β* = 60, *ϕ* = 4.7 eV, and *γ*_*SE*_ = 10^−4^ the FE/TA model and the UPL intersect to the left of the Paschen minimum. However, reducing *ϕ* to 3 eV shifts the intersection to the right of the Paschen minimum. Figure 4a shows ${(\bar{p}\bar{d})}_{int}/{(\bar{p}\bar{d})}_{PL,min}$ as a function of $\bar{p}$ for *β* = 60 and *γ*_*SE*_ = 10^−6^ considering *ϕ* = 2, 3.5, 5, and 6 eV $(\bar{\varphi }=0.0207,\,0.0362,\,0.0516,\,{\rm{and}}\,0.0620)$ and Fig. 4b shows ${(\bar{p}\bar{d})}_{int}/{(\bar{p}\bar{d})}_{PL,min}\,$as a function of $\bar{p}$ for various *β* at *γ*_*SE*_ = 10^−6^. We note that *ϕ* does not have a significant effect on the transition point until $\bar{p}\approx 2\times {10}^{-6}$ (which corresponds to 380 Torr). We also observe the same behavior for any small value of *γ*_*SE*_, such as *γ*_*SE*_ = 10^−6^ This is analogous to previous observations that *γ*_*SE*_ does not play a vital role until TA dominates breakdown (often occurring somewhere around atmospheric pressure)^26,50^. Figure 4b indicates that increasing *β* from 15 to 60 does not influence the transition point until $\bar{p}\approx 5\times {10}^{-6}\,$(950 Torr). Reducing *ϕ* can shift the transition to the left of the PL minimum at subatmospheric pressures, but changing *β* does not shift the transition to the left of the minimum until the pressure exceeds atmospheric pressure. Varying *γ*_*SE*_ yielded similar behavior on the intersection with PL as changing *β*.

## Conclusion

In summary, we applied a gas breakdown theory^26,27^ to assess experimental results for breakdown voltage at various pressures. Using *β* as a fitting parameter, we achieved excellent agreement between the exact numerical solution of the theory and the experimental results, and demonstrated that the analytic model differed from experiment by an average of 3.71%. We showed that experimental conditions, particularly electrode work function, can drive the intersection between the coupled FE/TA model and the UPL to the left or the right of the traditional Paschen minimum, providing a potential contributing factor determining whether *V*_*B*_ decreases with decreasing *pd* or an extended plateau occurs. Furthermore, the results showed that *β* and *γ*_*SE*_ have little influence on the location of the transition below atmospheric pressure, but *ϕ* has a greater influence. Future studies quantifying the change in work function^51^ with multiple breakdown events will further elucidate how breakdown behavior changes with constant gap distance. For example, one can envision an initial work function leading to a transition to the UPL to the right of the minimum, with subsequent breakdown events occurring to the left after electrode surface damage potentially decreases work function if it enhances surface roughness^51^. Future work quantifying how changes in work function due to surface roughness or chemical roughness^52^ effect the system and incorporating thermionic emission^53^ into the model will enhance the utility and completeness of the model across multiple operating regimes. A more thorough understanding of this behavior is vital to accurately predict breakdown behavior and electron emission overall.
